# A Multi‐functional Hybrid System Comprised of Polydopamine Nanobottles and Biological Effectors for Cartilage Repair

**DOI:** 10.1002/smll.202405979

**Published:** 2024-07-30

**Authors:** Min Hao, Younan Xia

**Affiliations:** ^1^ The Wallace H. Coulter Department of Biomedical Engineering Georgia Institute of Technology and Emory University Atlanta GA 30332 USA; ^2^ School of Chemistry and Biochemistry Georgia Institute of Technology Atlanta GA 30332 USA

**Keywords:** chondrogenic differentiation, nanobottles, programmable release, stem cells, tissue repair

## Abstract

Biological effectors play critical roles in augmenting the repair of cartilage injuries, but it remains a challenge to control their release in a programmable, stepwise fashion. Herein, a hybrid system consisting of polydopamine (PDA) nanobottles embedded in a hydrogel matrix to manage the release of biological effectors for use in cartilage repair is reported. Specifically, a homing effector is load in the hydrogel matrix, together with the encapsulation of a cartilage effector in PDA nanobottles filled with phase‐change material. In action, the homing effector is quickly released from the hydrogel in the initial step to recruit stem cells from the surroundings. Owing to the antioxidation effect of PDA, the recruited cells are shielded from reactive oxygen species. The cartilage effector is then slowly released from the nanobottles to promote chondrogenic differentiation, facilitating cartilage repair. Altogether, this strategy encompassing recruitment, protection, and differentiation of stem cells offers a viable route to tissue repair or regeneration through stem cell therapy.

## Graphical abstract

M. Hao and Y. Xia report the fabrication of a hybrid system comprises of polydopamine nanobottles embedded in a hydrogel for the stepwise release of biological effectors. This system is able to recruit stem cells from the surroundings, protect them from oxidative stress, and promote their chondrogenic differentiation, holding promise for cartilage repair and related applications.

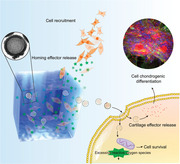

## 1 Introduction

Serving as the connective tissue between bones, articular cartilage plays an essential role in shock absorption and load‐bearing.^[^ ^1^ ^]^ An injury to cartilage is expected to trigger degenerative changes, ultimately leading to osteoarthritis.^[^ ^2^ ^]^ Among the numerous methods for treating articular cartilage injuries, stem cell therapy stands out as a viable approach owing to the self‐renewal and multi‐directional differentiation capacities of stem cells.^[^ ^3^ ^]^ Despite recent progress in applying stem cell therapy to the repair of cartilage injuries, various challenges persist. For example, the cartilage injury may compromise the precise control of local oxygen levels in vivo, further destabilizing the homeostasis of cellular mitochondria.^[^ ^4^ ^]^ When exposed to excessive reactive oxygen species (ROS), the cells are highly prone to apoptosis. As a result, the survival rate of stem cells within the microenvironment of the injury site poses a major obstacle to stem cell therapy. On the other hand, due to their potential of differentiating into osteoblasts, chondrocytes, etc.,  mesenchymal stem cells (MSCs) have emerged as a vital component for the treatment of cartilage injuries.^[^ ^5^ ^]^ However, it remains a challenge to recruit or deliver MSCs to the injury site, not mentioning the difficulty in directing their differentiation into chondrocytes.^[^ ^6^ ^]^

In addressing the survival issue, here we propose to introduce polydopamine (PDA) into the design of a scaffold owing to its excellent biocompatibility, controlled synthesis under mild conditions, and ease of functionalization for crafting specific structures.^[^ ^7^ ^]^ With abundant phenolic groups, PDA holds the potential to effectively scavenge ROS, thereby offering protection to the cells.^[^ ^8^ ^]^ As for cell recruitment and differentiation, we propose to introduce biological effectors such as stromal cell‐derived factor‐1 (SDF‐1) and kartogenin (KGN) into the scaffold. We choose to focus on these effectors because SDF‐1 can interact with its homologous receptor CXCR4 and thereby recruit stem cells to the injury site,^[^ ^9^ ^]^ while KGN can induce chondrogenic differentiation of the stem cells and protect cartilage by regulating the core binding factor‐β/runt‐related transcription factor 1 signaling pathway.^[^ ^10^ ^]^ By programming the release sequence of these effectors from PDA nanobottles, it is feasible to recruit and then regulate the differentiation of stem cells while protecting them from oxidative stress.

**Scheme 1:**
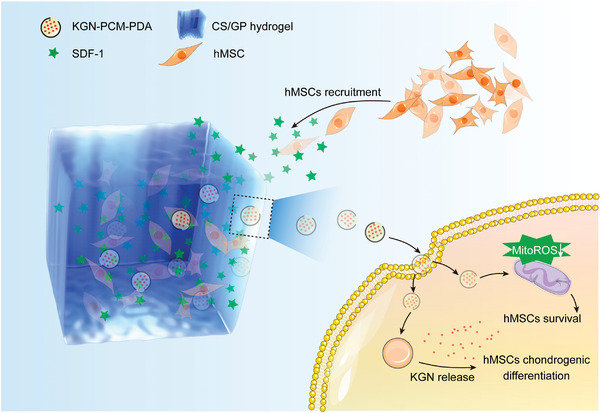
Schematic of a hybrid system comprised of PDA nanocarriers and a hydrogel for the recruitment, protection, and chondrogenic differentiation of hMSCs.

Specifically, we have demonstrated the fabrication of a hybrid system comprised of PDA nanobottles and a hydrogel for the programmed release of biological effectors (**Scheme** **1**). Similar to a conventional bottle, the PDA nanobottle features a hollow body and surface opening, allowing for convenient loading and release of effectors.^[^ ^11^ ^]^ With the help of a phase‐change material (PCM), cartilage effector KGN is loaded into PDA nanobottles to obtain KGN‐PCM‐PDA, which is then dispersed with homing effector SDF‐1 in a thermo‐sensitive hydrogel made of chitosan (CS) and β‐glycerophosphate (GP). Due to their capability to maintain the viability of encapsulated stem cells, CS‐based hydrogels show great potential for cartilage repair.^[^ ^12^ ^]^ In this hybrid system, SDF‐1 is released first from the hydrogel to recruit surrounding stem cells to the designated region. Benefiting from the antioxidative effect of PDA, the recruited cells are shielded from damage caused by oxidative stress. Meanwhile, KGN is gradually released from the PDA nanobottles, accelerating the chondrogenic differentiation of the stem cells. Altogether, the hybrid system encompasses the unique capabilities to recruit, protect, and regulate MSCs through the programmed release of different biological effectors, opening new prospects for the repair of cartilage injuries.

## 2 Results and Discussion

### 2.1 Fabrication of Nanobottles Filled with a Cartilage Effector

**Figure 1:**
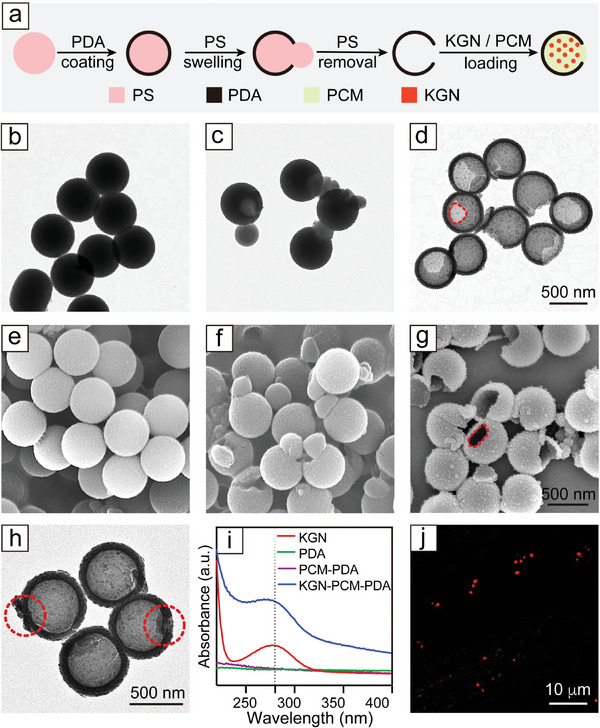
a) Schematic showing the fabrication of KGN‐PCM‐PDA. b–d) TEM images of the PS@PDA nanoparticles, PS‐PDA Janus nanoparticles, and PDA nanobottles. The panels share the same scale bar. e–g) SEM images of the PS@PDA nanoparticles, PS‐PDA Janus nanoparticles, and PDA nanobottles. The panels share the same scale bar. h) TEM image of the KGN‐PCM‐PDA particles. i) UV–vis spectra recorded from KGN, PDA, PCM‐PDA, and KGN‐PCM‐PDA. j) Fluorescence micrograph of the RhB‐PCM‐PDA particles.

**Figure** **1**a shows a schematic of the process for the fabrication of PDA nanobottles filled with KGN, a cartilage effector. It started with the polymerization of dopamine on the surface of polystyrene (PS) beads to obtain a uniform, conformal shell. Figure 1b–g and Figure S1 (Supporting Information) show transmission electron microscopy (TEM) and scanning electron microscopy (SEM) images of the sample at various stages of fabrication. The PS@PDA core‐shell nanoparticles had a uniform diameter of 530 nm. When mixed with a toluene/water emulsion, the PS core was swollen to generate pressure against the PDA shell. Once the pressure surpassed a threshold, a single opening was created in the shell, allowing the swollen PS to escape through the hole and thereby releasing the pressure for the generation of PS‐PDA Janus nanoparticles. After dissolving the PS with tetrahydrofuran, we obtained PDA nanobottles with a surface opening of ca. 230 nm, together with a uniform diameter of ca. 550 nm. Subsequently, the PDA nanobottles were mixed with the cartilage effector in methanol, together with a PCM made of lauric acid and stearic acid. After removing the unloaded materials by washing and centrifugation, the sample was dispersed in water to solidify the PCM to produce PDA nanocarriers filled with a cartilage effector (Figure 1h). At this point, the opening on the surface of the nanoparticles was still visible under TEM, as indicated by the dashed red circles. The sample exhibited a characteristic absorption peak at 280 nm in the ultraviolet‐visible (UV–vis) spectrum (Figure 1i), further confirming the successful loading of the cartilage effector, together with PCM, into PDA nanobottles. To visualize the payload, we replaced the cartilage effector with rhodamine B (RhB) to obtain RhB‐PCM‐PDA. As shown by the fluorescence micrograph in Figure 1j, the dye was indeed successfully loaded to give a uniform distribution of red particles.

### 2.2 Fabrication and Characterizations of the Hybrid System

**Figure 2:**
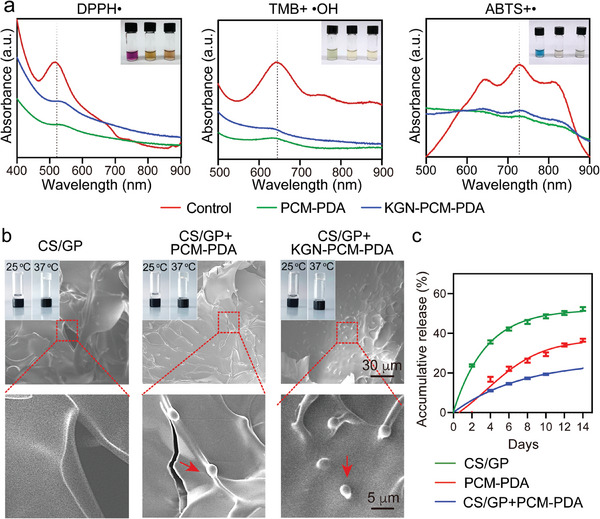
a) UV–vis spectra of the radicals incubated with PDA nanocarriers. Control: radicals only. The insets are photographs of control, PCM‐PDA, and KGN‐PCM‐PDA arranged from left to right. b) SEM images of the hydrogel and hybrid systems, with the insets showing photographs of the samples at 25 and 37 °C, respectively. c) Accumulative release of fluorescein isothiocyanate (FITC) from the CS/GP hydrogel, PCM‐PDA, and CS/GP+PCM‐PDA in phosphate‐buffered saline (PBS) solution, respectively, with 50% of the PBS solution being replaced at each sampling point every 2 days.

ROS primarily encompasses superoxide anion, ·OH, and hydrogen peroxide (H₂O₂).^[^ ^13^ ^]^ With an abundance of phenolic groups, PDA nanomaterials can readily generate an electron‐donating state to effectively suppress ROS through a dynamic catechol/quinone redox system.^[^ ^14^ ^]^ To this end, we evaluated the impact of PDA nanocarriers on the concentration of ROS by leveraging well‐known free radical reactions (**Figure** **2**a; Figure S2, Supporting Information). Specifically, as a stable free radical, 1,1‐diphenyl‐2‐picryl‐hydrazyl radical (DPPH) exhibited a characteristic peak at 517 nm (the control group).^[^ ^15^ ^]^ Upon introduction of PDA nanobottles capable of donating an electron pair to exert the antioxidant role, the solution gradually faded from the deep purple color, with the absorption peak at 517 nm diminishing and eventually disappearing. Tetramethylbenzidine (TMB) functions as an indicator for ·OH, changing from colorless to blue‐green in the presence of ·OH.^[^ ^16^ ^]^ The absorption peak of TMB at 652 nm decreased and the color of the solution lightened when PDA nanobottles were introduced into the system. The cationic radical 2,2′‐Azinobis‐3‐ethylbenzthiazoline‐6‐sulphonate (ABTS+·) exhibits a blue color, together with an absorption peak at 734 nm.^[^ ^17^ ^]^ Again, the introduction of PDA nanobottles decreased the absorption peak intensity as the solution faded in color. These results from free radical reactions demonstrated the capability of PDA nanocarriers to scavenge ROS, establishing the basis for cell protection.

We dispersed the loaded nanobottles in the GS/GP hydrogel to yield groups of CS/GP+PCM‐PDA and CS/GP+KGN‐PCM‐PDA. As reported in the literature, the thermo‐sensitive properties of the hydrogel can be attributed to the decrease in electrostatic repulsion among the protonated amino groups above a critical temperature, along with the formation of intramolecular hydrogen bonds.^[^ ^18^ ^]^ After incubating the dispersion in a vial at 25 or 37 °C for 1 min, we inverted the vial (inset of Figure 2b). At 25 °C, the mixture existed in a sol state and was able to flow toward the neck of the vial. In contrast, the mixture transitioned into a gel state at 37 °C, sticking to the bottom of the vial. The introduction of PDA nanocarriers showed no obvious influence on the thermo‐sensitive properties of the hydrogel. The SEM image taken from a freeze‐dried sample confirmed the successful integration of PDA nanocarriers into the hydrogel (Figure 2b), as indicated by the red arrows, yielding a composite. In addition, we dispersed the PDA nanocarriers pre‐loaded withFITC into the hydrogel, followed by 3D analysis using laser confocal microscopy (Figure S3, Supporting Information). The data confirmed that the nanocarriers were uniformly distributed in the hydrogel. We also substituted KGN with FITC and examined its release profiles from the different systems (Figure 2c; Figure S4, Supporting Information). The fluorescent molecule was gradually released from the hydrogel and PCM‐PDA nanocarriers over two weeks. Specifically, after 2 days of incubation, the accumulative release of the payload from the hydrogel was much greater than those from the other two samples. The quick release from the hydrogel matrix was well‐suited for the proposed function of the homing effector. After 14 days of culture, the accumulative release of the payload from the hybrid system (ca. 22.4%) was lower than those of the other two samples, which were ca. 52.3% and 36.5%, respectively. Such a slow and sustained release of the cartilage effector holds the key to its prolonged efficacy.

### 2.3 Biocompatibility of the PDA Nanobottles and the Hybrid System

**Figure 3:**
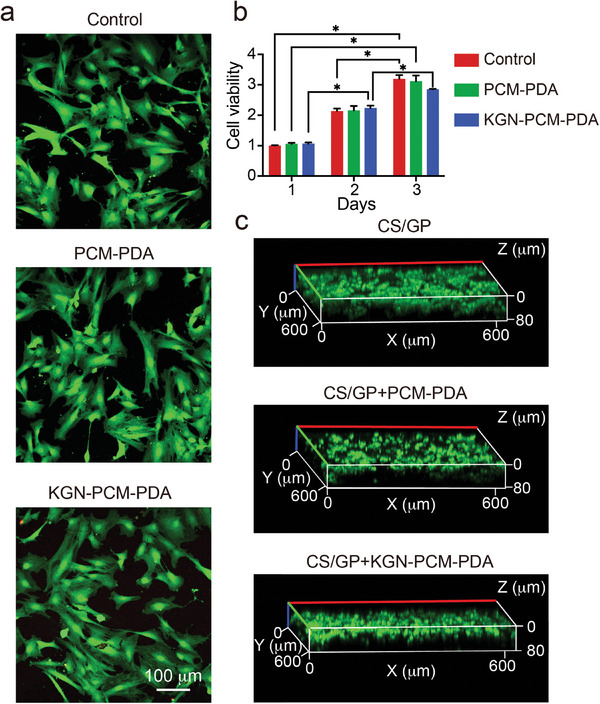
a) Confocal micrographs after live/dead staining of the hMSCs cultured with the two PDA nanocarriers for 48 h. The live and dead cells are stained green and red, respectively. b) CCK8 assay after hMSCs cultured with the two PDA nanocarriers for 1, 2, and 3 days. Control group: hMSCs only. The data are normalized to the control group on day 1 and are presented as mean ± standard deviation (SD) (*n* = 3). Two‐way analysis of variance (ANOVA) with Tukey's multiple comparisons test (^*^ *p* < 0.05). c) Z‐stack confocal micrographs after live/dead staining of the hMSCs cultured in the hydrogels for 48 h.

To evaluate the biocompatibility, we performed live/dead staining of cells after culture with the PCM‐PDA or KGN‐PCM‐PDA sample for 48 h (**Figure** **3**a).^[^ ^19^ ^]^ We used human mesenchymal stem cells (hMSCs) for all tests. Calcein AM stained live cells in green while ethidium homodimer‐1 stained dead cells in red. The cells cultured with both systems remained alive, with only a few dead cells being observed. Whether the cells were cultured with the PDA nanocarriers or not, they shared a similar survival rate of exceeding 95% (Figure S5, Supporting Information). In addition, we assessed the influence of PDA nanocarriers on cell proliferation at 1, 2, and 3 days through a cell counting kit 8 (CCK8) assay (Figure 3b). Again, the cell viability was essentially the same across all samples, and it gradually increased as the cultured time was increased from 1 to 2 and 3 days. This result indicated that the involvement of PDA nanocarriers did not affect the proliferative capacity of the cells.

Furthermore, we mixed the cells with the hydrogel, in the absence/presence of the PDA nanocarriers, at 25 °C and then increased the temperature to 37 °C. The cells were encapsulated within the hydrogel matrix in situ to form a uniform, 3D distribution. The cells within all three samples exhibited a spherical shape (Figure 3c; Figure S6, Supporting Information), and most of them remained alive, with a cell survival rate exceeding 95% (Figure S5, Supporting Information). The negative control based upon the CS/GP hydrogel only showed no obvious fluorescence signal (Figure S7, Supporting Information). These results demonstrated that the hydrogel, as a medium for cell encapsulation, also exhibited good biocompatibility. Taken together, the hybrid system possessed good biocompatibility, ensuring natural proliferation for the cells.

### 2.4 Internalization of and Protection from the PDA Nanocarriers

**Figure 4:**
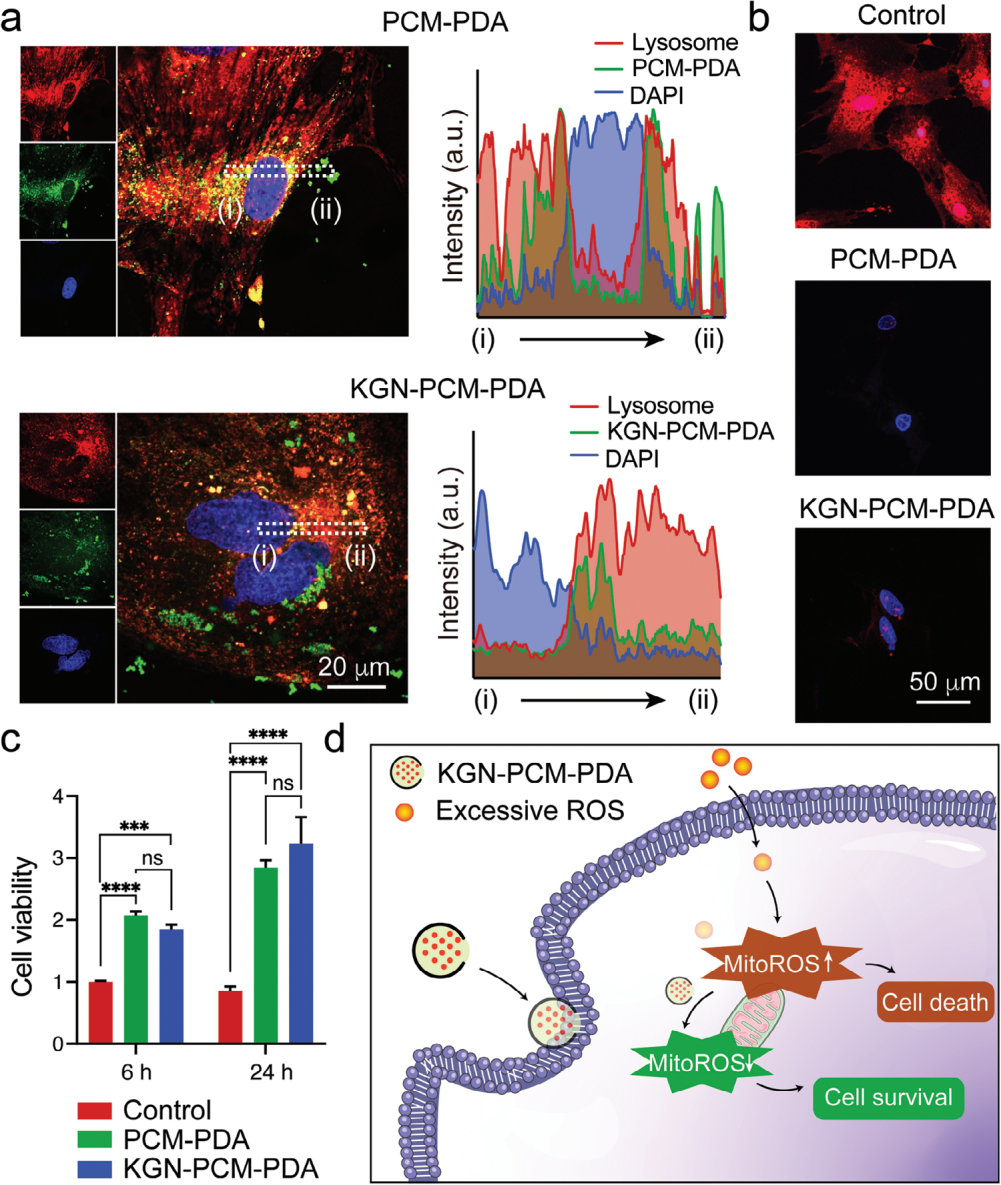
a) Confocal micrographs of hMSCs after culture with two types of PDA nanocarriers (green) for 12 h, followed by lysosome (red) and 4′,6‐diamidino‐2‐phenylindole (DAPI, blue) staining, and plots of the fluorescence intensities. b) Confocal micrographs of hMSCs culture with two types of PDA nanocarriers for 3 h, followed by MitoSOX (red) and DAPI (blue) staining. c) CCK8 assay after 6 and 24 h of culture. *n* = 3. One‐way ANOVA with Tukey's multiple comparisons test (^**^ *p* < 0.01 and ^****^ *p* < 0.0001). The data are normalized to the control group at 6 h. d) Schematic showing the internalization of KGN‐PCM‐PDA for cell protection against excessive ROS.

To explore the interactions between cells and PDA nanocarriers, including PCM‐PDA and KGN‐PCM‐PDA, we characterized the samples of different culture time using confocal microscopy. In general, the internalized nanomaterials should be transported to the lysosome of a cell,^[^ ^20^ ^]^ so we focus on the correlation between the locations of cell lysosome and PDA nanocarrier by labeling lysosome with LysoTracker for red fluorescence while marking PDA with FITC for green fluorescence. After 6 h of culture, there is no apparent co‐localization between their positions, as demonstrated by the plots of fluorescence intensities from region (i) to (ii) (Figure S8a, Supporting Information). At this time point, the nanocarriers were primarily distributed around the cells. It is worth noting that the positions of red and green fluorescence overlapped in both two groups after incubation for 12 h (**Figure** **4**a), which was further verified by the plots of fluorescence intensities from region (i) to (ii). This result suggested that the PDA nanocarriers were internalized by cells after 12 h of culture. Similarly, after 24 h of culture, the co‐localization between the lysosome and nanocarrier remained (Figure S8b, Supporting Information), further confirming the internalization of the nanocarrier by the cell.

Internalization is a general pathway for the nanoparticles to impact cell survival, migration, and differentiation.^[^ ^21^ ^]^ In the case of elevated oxidative stress, the mitochondrial homeostasis of a cell is prone to disruption, leading to cellular apoptosis.^[^ ^22^ ^]^ As we discussed in Figure 2a, PDA nanobottles possessed antioxidant capability owing to the presence of phenolic groups. To this end, we cultured the cells exposed to H_2_O_2_ with PDA nanocarriers for 3 h, followed by an analysis of the cellular mitochondrial superoxide (Figure 4b; Figure S9, Supporting Information). We used MitoSOX as a probe to analyze the mitochondria of live cells due to its oxidization by mitochondrial superoxide.^[^ ^23^ ^]^ Notably, in the presence of the PDA nanobottles, the expression of superoxide was reduced to levels of ca. 22 and 32% of the control group, respectively. We also evaluated cell viability after 6 and 24 h of culture (Figure 4c). After 6 h of culture, the viabilities of cell culture with nanocarriers were ca. 2.1‐ and 1.9‐folds as high as that of the control group, respectively. After 24 h of culture, the viability increased by ca. 3.3‐ and 3.8‐folds, respectively, relative to that of the control group. The results indicated that the PDA nanocarriers could indeed improve the viability of cells under oxidative stress. Taken together, PDA nanocarriers could be internalized by cells (Figure 4d), proving a mechanism for cell protection and fate regulation. Meanwhile, PDA nanocarriers could resist oxidative stress to maintain cellular mitochondrial homeostasis, thereby ensuring cell viability.

### 2.5 Chondrogenic Differentiation Induced by the Cartilage Effector

**Figure 5:**
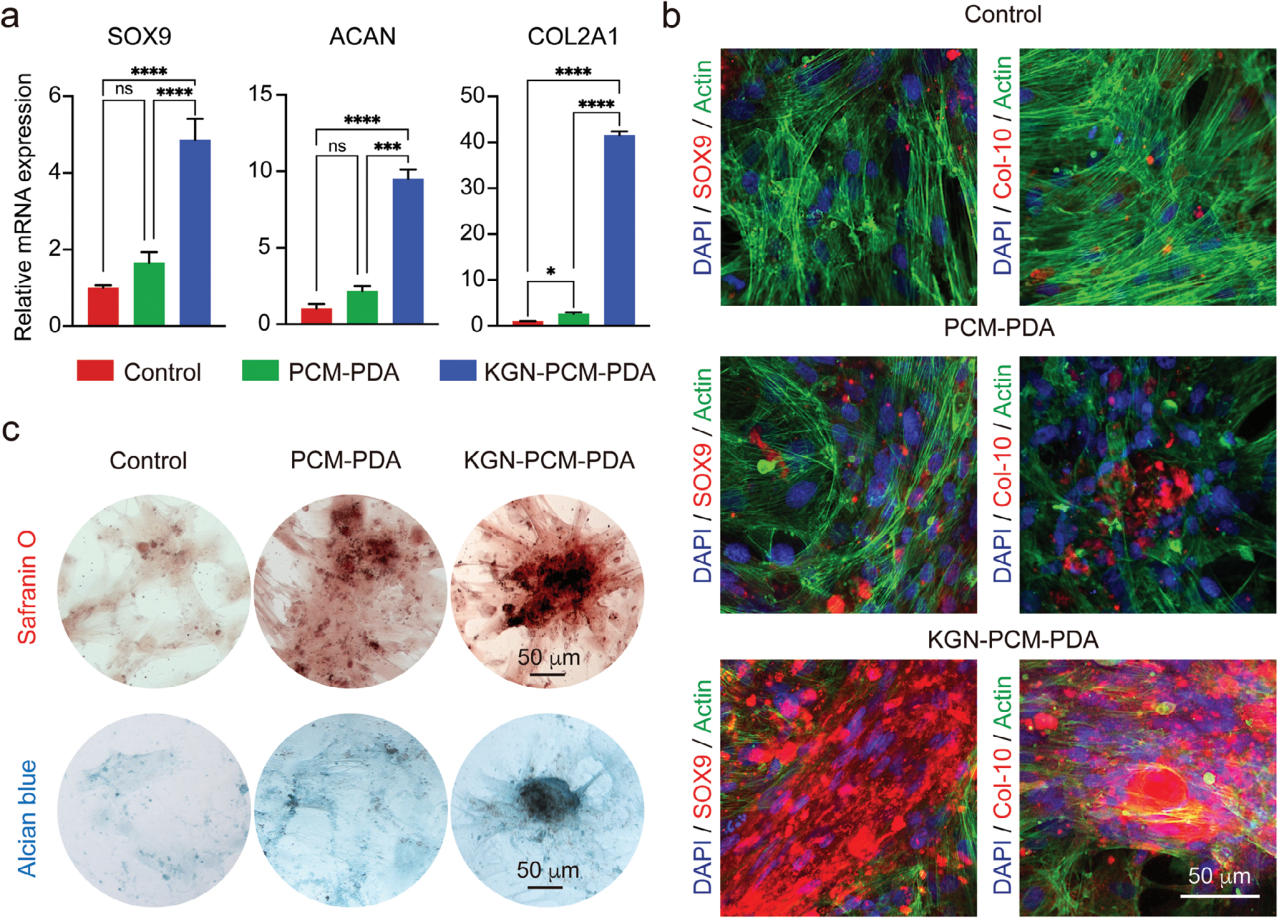
a) RT‐qPCR analysis of hMSCs culture with two types of PDA nanocarriers for 14 days. *n* = 3. One‐way ANOVA with Tukey's multiple comparisons test (ns, no significant; ^***^ *p* < 0.001 and ^****^ *p *< 0.0001). b) Confocal micrographs of hMSCs culture with two types of PDA nanocarriers for 21 days, followed by Actin/SOX9/DAPI and Actin/Col‐10/DAPI staining. Actin staining was just used to label the distribution of cells. c) Optical micrographs of safranin O and alcian blue staining for hMSCs after 21 days of culture.

Next, we assessed the differentiation and maturation of hMSCs after culture with the PDA nanocarriers for 14 days via reverse transcription‐polymerase chain reaction (RT‐qPCR) analysis (**Figure** **5**a). Specifically, we used SRY‐box transcription factor 9 (SOX9) as a major transcription marker for early chondrogenesis.^[^ ^24^ ^]^ Maintained at a high level in fully differentiated cartilage, it guides the generation of aggrecan (ACAN) and collagen type II alpha 1 chain (COL2A1).^[^ ^25^ ^]^ The mRNA level of SOX9 in samples involving KGN was ca. 4.9‐ and 3.0‐folds higher than those of the other two groups, respectively. As a primary component of the extracellular matrix in cartilage tissue,^[^ ^26^ ^]^ its mRNA also exhibited high levels in samples involving KGN, being ca. 9.5‐ and 4.4‐folds higher than the other two groups, respectively. Serving as a fibrillar collagen in cartilage, COL2A1 is commonly considered a specific marker for chondrogenic differentiation.^[^ ^27^ ^]^ Similarly, the mRNA level of COL2A1 in samples involving KGN was ca. 41.6‐ and 15.8‐folds higher than the other two groups, respectively. Collectively, the mRNA levels of cartilage‐specific markers demonstrated that the KGN released from PDA nanobottles has the capability to promote the chondrogenic differentiation of hMSCs.

After 21 days of chondrogenic differentiation, immunofluorescence staining was performed to fluorescently visualize SOX9 and collagen type X alpha 1 chain (Col‐10), which are the early and late markers of chondrogenesis, respectively (Figure 5b; S10, Supporting Information).^[^ ^28^ ^]^ The samples involving KGN expressed more SOX9 (ca. 67.3% in average fluorescence intensity) than the other two groups (ca.16.2% and 34.8%, respectively). The expression of Col‐10 was similar to SOX9, with a higher level observed in the sample involving KGN, displaying an average fluorescence intensity of ca.1.7‐ and 7.8‐folds greater than the other two groups, respectively. The results further confirmed that the KGN effector released from PDA nanobottles accelerated the chondrogenic differentiation of hMSCs. In the 3D microenvironment of a hydrogel, the samples involving KGN also expressed more cartilage‐specific genes and proteins (Figure S11, Supporting Information), suggesting that the chondrogenesis strategy remains effective in a 3D microenvironment.

Glycosaminoglycans (GAGs) are crucial components of cartilage.^[^ ^29^ ^]^ The maintenance of chondrocyte phenotype and cartilage tissue relies on the synthesis of sufficient GAGs.^[^ ^30^ ^]^ We therefore evaluated the deposition and distribution of GAGs by labeling samples with safranin O and alcian blue dyes after 21 days of culture (Figure 5c). A higher level of GAGs was observed in samples involving KGN than the other two groups, indicating better maintenance of cartilage tissue. These data demonstrated that the KGN effector released from PDA nanobottles was able to induce cell chondrogenic differentiation and facilitate the deposition of the cartilage matrix.

### 2.6 Cellular Responses to the Released Biological Effectors

**Figure 6:**
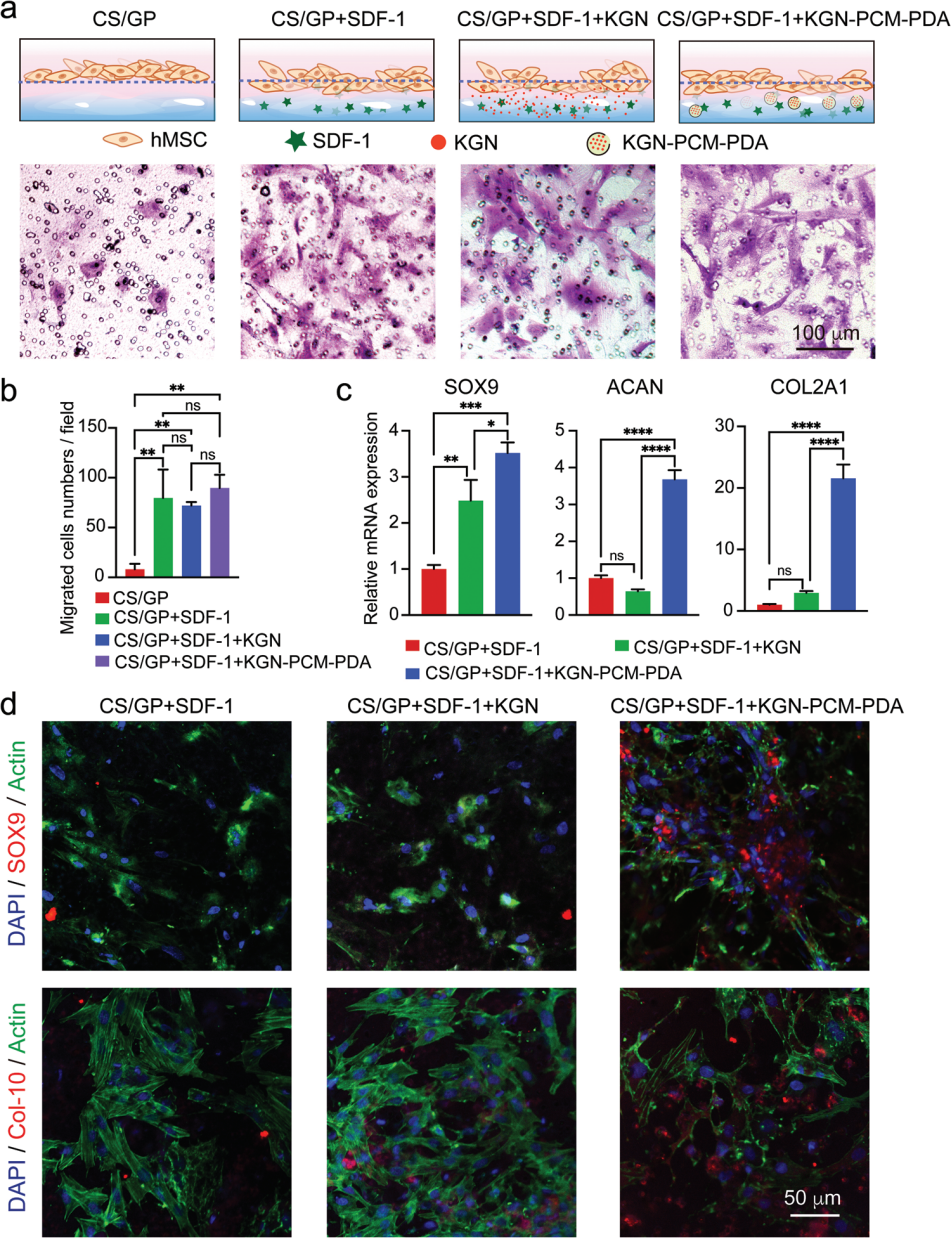
a) Schematic of the Transwell cell migration assay involving the use of a microporous membrane for cell culture and optical micrographs of the hMSCs residing on the basal side of the microporous membrane after culture for 12 h, followed by crystal violet staining. b) Statistics of cell migration analysis after 12 h of culture. *n* = 3. One‐way ANOVA with Tukey's multiple comparisons test (^**^ *p* < 0.01). c) RT‐qPCR analysis of hMSCs on the underside of the microporous membrane after 14 days of culture. *n* = 3. One‐way ANOVA with Tukey's multiple comparisons test (^*^ *p* < 0.05, ^**^ *p* < 0.01, ^***^ *p* < 0.001, and ^****^ *p* < 0.0001). d) Confocal micrographs after Actin/SOX9/DAPI and Actin/Col‐10/DAPI staining of hMSCs on the underside of the microporous membrane cultured for 21 days.

To understand the influence of the programmed release of biological effectors from the hybrid system on cells, we utilized a Transwell system to directly observe cellular responses to the hybrid system in vitro.^[^ ^31^ ^]^ Briefly, the Transwell system is divided into two chambers by a microporous membrane, with cells being seeded on the apical side of the membrane. The bottom chamber was loaded with various types of hydrogel samples in four groups (**Figures** **6**a and S12, Supporting Information). After 12 h of culture, the cells on the apical side of the membrane were removed with a cotton swab and the cells on the basal side were stained with crystal violet and DAPI.^[^ ^32^ ^]^ More stained cells were observed in samples involving homing effector SDF‐1 than in the case of plain CS/GP hydrogel. A quantitative analysis indicated that the number of migrated cells in samples involving SDF‐1 was increased by ca. 10.0, 9.0, and 11.2 folds, respectively, relative to that of the CS/GP control group (Figure 6b). The data demonstrated that the homing effector effectively attracted cells to the hydrogel region, potentially playing a crucial role in recruiting hMSCs.

Based on the functionality of the homing effector, we further evaluated the role of KGN on cells by examining the cartilage‐specific gene expression for samples involving the homing effector after 14 days of culture (Figure 6c). During the culture, the medium was partially replaced every 24 h to simulate the flow of bodily fluid in vivo. For the sample involving KGN‐PCM‐PDA nanocarriers, the mRNA level of the transcription factor SOX9 was ca. 3.5‐ and 1.4‐folds higher than the other two groups, respectively. It demonstrated that cartilage effector KGN, especially encapsulated in nanocarriers, was more effective in promoting cell chondrogenic differentiation. Besides, the cartilage‐specific markers of ACAN and COL2A1 were expressed later than SOX9. The mRNA level of ACAN in the sample involving KGN‐loaded PDA nanocarriers was ca. 5.7‐ and 3.7‐folds higher than the other two groups, together with ca. 7.4‐ and 21.6‐fold enhancement in COL2A1 mRNA expression, respectively. These data suggested that the gradual and sustained release of cartilage effector from the nanobottles in a hydrogel was more conducive to cell chondrogenic differentiation. Figure 6d and Figure S13 (Supporting Information) depict the visualization of cartilage‐specific markers and GAG synthesis, respectively, for samples after 21 days of culture.^[^ ^33^ ^]^ The sample involving KGN‐PCM‐PDA possessed the highest expression of cartilage‐specific markers of SOX9 and Col‐10, as well as GAG synthesis, among the three groups, confirming the crucial role of the slow release of cartilage effector in cell chondrogenic differentiation. Taken together, the fast release from the hydrogel in the early stage was conducive to homing effector in recruiting stem cells. In contrast, the slow and sustained release of cartilage effector from the nanocarriers embedded in the hydrogel was more beneficial for long‐term chondrogenic differentiation.

## 3 Conclusion

We have demonstrated a hybrid system comprised of PDA nanobottles and a hydrogel to stepwise release biological effectors for the recruitment and protection of hMSCs while promoting their chondrogenic differentiation. Specifically, cartilage and homing effectors are encapsulated in the nanobottles and hydrogel, respectively, to align with their stages of operation. Owing to the fast‐release profile of payload from hydrogel, the homing effector is swiftly released to recruit stem cells within the first 12 h. The recruited stem cells are protected from oxidative stress owning to the antioxidative properties arising from the phenolic groups of PDA. Meanwhile, the cartilage effector slowly released from the nanobottles embedded in the hydrogel promotes chondrogenic differentiation. This stepwise release strategy holds promise for disease therapy that requires the sequential action of multiple effectors.

## Conflict of Interest

The authors declare no conflict of interest.

## Author Contributions

M.H. and Y.X. conceived the project. M.H. performed experiments and analyzed data. M.H. and Y.X. wrote and revised the manuscript. Y.X. provided the resources and supervised the project.

## Supporting information

Supporting Information

## Data Availability

The data that support the findings of this study are available from the corresponding author upon reasonable request.
