# Supplementary material for: A Multi‐functional Hybrid System Comprised of Polydopamine Nanobottles and Biological Effectors for Cartilage Repair
Source: Small. 2024 Jul 30;20(50):2405979. doi: 10.1002/smll.202405979 (PMC11636171; doi:10.1002/smll.202405979)
Supplement: Supplementary file 1 — Supporting Information [file SMLL-20-2405979-s001.docx]

**Supporting Information**

**A Multi-functional Hybrid System Comprised of** **Polydopamine Nanobottles and Biological Effectors for Cartilage Repair**

Min Hao and Younan Xia*

Prof. Dr. Y. Xia, Dr. M. Hao

The Wallace H. Coulter Department of Biomedical Engineering

Georgia Institute of Technology and Emory University

Atlanta, GA 30332 (USA)

E-mail: [younan.xia@bme.gatech.edu](mailto:younan.xia@bme.gatech.edu)

Prof. Y. Xia

School of Chemistry and Biochemistry

Georgia Institute of Technology

Atlanta, GA 30332 (USA)

**Experimental Section**

*Chemicals and Materials*

Dopamine hydrochloride, sodium dodecyl sulfate (SDS, 99%), poly(allylamine hydrochloride) (Mw≈50,000), toluene (99.5%), tetrahydrofuran (THF, 99.9%), methanol (99.8%), lauric acid, stearic acid, fluorescein isothiocyanate (FITC), rhodamine B (RhB), chitosan (CS), glycerophosphate (GP), dexamethasone, ascorbic acid, potassium persulfate, Triton X-100, bovine serum albumin (BSA), safranin O and alcian blue were purchased from Sigma-Aldrich. Polystyrene (PS) beads of 500 nm in diameter were obtained from Polysciences. 2,2’-azinobis-3-ethylbenzthiazoline-6-sulphonate (ABTS), 1,1-diphenyl-2-picrylhydrazyl free radical (DPPH), ferrocene-carboxylic acid, tetramethylbenzidine (TMB), calcein AM, ethidium homodimer-1 (EthD-1), and paraformaldehyde were purchased from Thermo Fisher Scientific. Cell counting kit 8 (CCK8) was ordered from Dojindo. Alpha minimum essential medium (α-MEM), advanced dulbecco’s modified eagle medium/ham’s F-12 (DMEM/F-12) medium, fetal bovine serum (FBS), and penicillin/streptomycin were purchased from Gibco. MitoSOX™ mitochondrial superoxide indicator, TRIzol reagent, and anti-SOX9 antibody (Mouse monoclonal, 1:500, 14-9765-82) were purchased from Invitrogen. QuantiTect reverse transcription kit was obtained from QIAGEN. Anti-COL-10 (Mouse monoclonal, 1:1000, ab49945) and goat anti-mouse IgG H&L Alexa Fluor^®^ 594 (1:1000, ab150116) antibodies were purchased from Abcam. CoraLite^®^ Plus 488-Phalloidin (1:200, PF00001) was from Proteintech.

*Preparation of* *PS-polydopamine (PDA) Core-shell Nanoparticles*

We prepared PS@PDA core-shell nanoparticles by following our previous method.^[1]^ In detail, 0.5 mL of the PS beads were washed with deionized (DI) water three times and then dispersed in 10 mL of Tris-HCl buffer (10 mM, pH=8.5) containing dopamine hydrochloride (1 mg/mL) overnight. The nanoparticles were collected by centrifugation and dispersed in DI water for further use.

*Fabrication of PDA Nanobottles*

The core-shell nanoparticles were mixed with 10 mL of 1% (v/v) toluene/water emulsion involving 1% (w/v) SDS, followed by shaking at 300 rpm at 25 °C for 6 h. After introducing 20 mL of ethanol (anhydrous, ≥99.5%) to quench the swelling, the products were collected through centrifugation (6,000 rpm, 5 min) and washed with ethanol. We then incubated the Janus nanoparticles with THF on a shaker (300 rpm) overnight to remove the PS. After washing with THF three times, PDA nanobottles with a surface opening were obtained.

*Filling PDA Nanobottles with a Phase-change Material (PCM) and a Molecular Payload*

Typically, 0.4 g of lauric acid and 0.1g of stearic acid (serving as the PCM), together with 50 μL of 100 μg/mL kartogenin (KGN) were dissolved in 0.5 mL of methanol (≥99.5%). Subsequently, the above solution was mixed with 50 µL ethanol (anhydrous, ≥99.5%) containing 10 mg of the PDA nanobottles, followed by shaking (300 rpm) at 40 °C for 2 h to fill the nanobottles with PCM and KGN. The product was collected through centrifugation (6000, 5min) and washed with 75% methanol three times. Then, 1 mL of DI water was introduced to solidify the PCM and thus keep KGN in the nanobottles. In similar attempts, we substituted KGN with FITC or RhB to obtain FITC-PCM-PDA or RhB-PCM-PDA.

*Preparation of CS/GP Thermo-sensitive Hydrogel*

2% (w/v) CS and 50% (w/w) GP solution were prepared by dissolving CS powder in 0.1 M acetic acid and GP in DI water, respectively. Then, 500 μL of 2% (w/v) CS solution was mixed with 100 μL of 50% (w/w) GP solution to form a hydrogel. We observed the thermosensitive properties of the hydrogel by placing it at 25 °C and 37 °C.

*Free Radical Scavenging Assay*

In the case of ABTS method, the free radical was generated after incubating 3 mg of ABTS and 1 mg of potassium persulfate in 500 μL of DI water for 12 h. Afterward, a mixture of 5 μL of free radical ABTS solution, PDA nanocarriers (10 μL, 20 mg/mL), and 985 μL of DI water was incubated in the dark for 15 min. In the case of the DPPH method, PDA nanocarriers (10 μL, 20 mg/mL) were incubated with DPPH (0.1 mM, 1 mL) in the dark for 30 min. In the case of the TMB method, PDA nanocarriers (10 μL, 20 mg/mL) were incubated with TMB, ferrocene-carboxylic acid (0.1 mM), and H_2_O_2_ (1 mM) for 15 min. Then, the absorbances of the above samples were recorded using a UV-vis spectrometer.

*Cell Culture and Encapsulation*

The human mesenchymal stem cells (hMSCs) were obtained from a commercial source (Lonza, Basel, Switzerland) and were recovered from cryopreservation. After being recovered from cryopreservation, the cells were cultured until the third generation in α-MEM supplemented with 10% FBS and 1% penicillin/streptomycin prior to being used. The culture medium was replaced by DMEM/F-12 with 10% FBS, 1% penicillin/streptomycin, 100 nM dexamethasone, 50 μg/mL ascorbic acid, and 1% insulin-transferrin-selenium for cell chondrogenic differentiation. During the culture, the medium was replaced every 2 days.

*Cell Live/dead Staining*

After 48 h of culture, the cell culture medium was replaced with serum-free α-MEM containing calcein AM and EthD-1 according to the instruction from the manufacturer, followed by incubating the samples at 37 °C for 20 min. Then, the samples were washed with PBS three times and analyzed using a confocal laser scanning microscope (CLSM).

*CCK8 Assay*

After cell culture for 1, 2, and 3 days, the cell culture medium was replaced by serum-free α-MEM containing 10% CCK8 solution and cultured at 37 °C for 1 h. After that, the samples were analyzed using a microplate reader at a wavelength of 450 nm.

*Mitochondrial Superoxide Live Cell Tracking*

We incubated cells with the medium containing MitoSOX™ mitochondrial superoxide indicator for 20 min according to the manufacturer’s instructions. After that, the living cells were analyzed using CLSM.

*Reverse Transcription-polymerase Chain Reaction (RT- qPCR) Analysis*

Briefly, we incubated cells with a TRIzol reagent to extract the total RNA of the cells. Then, the reverse transcription of total RNA into cDNA was performed according to the instruction of QuantiTect Reverse Transcription Kit. Finally, we used the StepOnePlus™ Real-Time PCR System to gain the C_T_ value of genes. The primer sequences are listed in Supplementary Table 1.

*Immunofluorescence*

Firstly, the samples were fixed with 4% paraformaldehyde for 15 min, followed by 0.1% Triton X-100 for 5 min. After blocking with 1% BSA for 30 min, the samples were incubated with primary antibodies of anti-SOX9 (Mouse monoclonal, 1:500) or anti-COL-10 (Mouse monoclonal, 1:1000) antibodies overnight, followed by the secondary antibody of Goat anti-mouse IgG H&L Alexa Fluor^®^ 594 (1:1000) incubated for 1 h. After that, the samples were incubated with CoraLite^®^ Plus 488-Phalloidin (1:200) for 20 min and DAPI for 5 min. Finally, the samples were evaluated using CLSM.

*Crystal Violet Staining*

The Transwell insert with an 8-µm polyester microporous membrane was used for cell migration assay. Briefly, the cells were seeded on the apical side of the membrane while various hydrogel samples were introduced into the bottom chamber. In the crystal violet staining, the cells on the apical side of the membrane were removed by cotton swabs, and the cells residing on the basal side of the membrane were fixed with 4% paraformaldehyde at 25 ℃ for 15 min. Then, the samples were incubated with 0.1% crystal violet solution for 15 min. After washing with PBS three times, the samples were evaluated using an optical microscope.

*Safranin O/Alcian Blue Staining*

After 21 days of culture, the cells were fixed with 4% paraformaldehyde for 15 min, followed by staining with 0.1 % (w/v) safranin O or 1% w/v alcian blue solution for 30 min. After washing with PBS three times, the samples were analyzed using an optical microscope.

*Instrumental Characterization*

The morphology and physical properties of the samples were analyzed using SU8230 SEM (Hitachi, Japan) and HT7700 TEM (Hitachi, Japan). The fluorescence micrographs from samples were obtained using a CLSM (Zeiss Co., Germany). The optical micrographs were captured using a CTR6000 microscope (Leica, Germany). The UV-vis spectrum was recorded on Cary 60 UV-vis (Agilent Technologies, USA).

*Statistical Analysis*

Data were presented as the mean ± standard deviations (SD). Statistical analyses were performed with GraphPad Prism 10 and Image J. Statistical significance differences were calculated with one-way (one independent variable) or two-way (two independent variables) ANOVA with Tukey’s comparison test and defined with p < 0.05. The sample numbers (n) were listed in the figure captions.

**Table 1.** The sequences of primers for RT-qPCR.

| Gene | Forward primers (5’-3’) | Reverse primers (5’-3’) |
| --- | --- | --- |
| β-actin | CATGTACGTTGCTATCCAGGC | CTCCTTAATGTCACGCACGAT |
| SOX9 | GGCAAGCTCTGGAGACTTCTG | CCCGTTCTTCACCGACTTCC |
| ACAN | AGTGCACAGAGGGGTTTGTC | CGTTTGTAGGTGGTGGGGTC |
| COL2A1 | GGGATCGTGGTGACAAAGGT | CTGGGCAGCAAAGTTTCCAC |


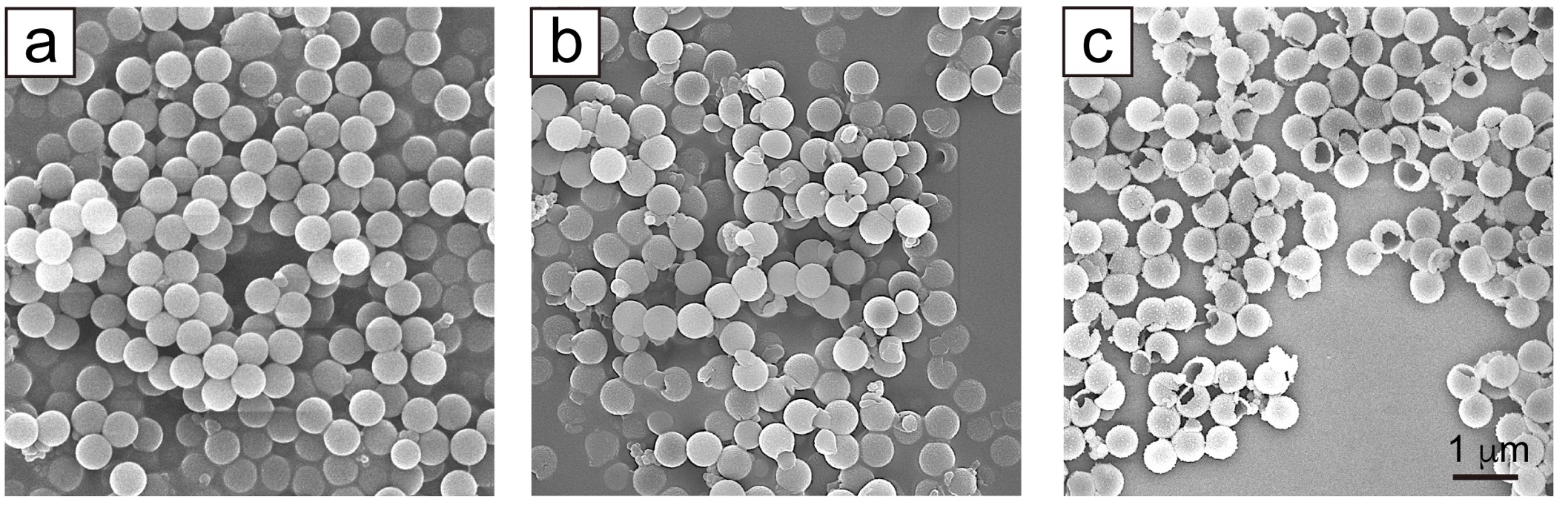


**Figure S1.** SEM images of the (a) PS@PDA core-shell nanoparticles, (b) PS-PDA Janus nanoparticles, and (c) PDA nanobottles. The panels share the same scale bar.


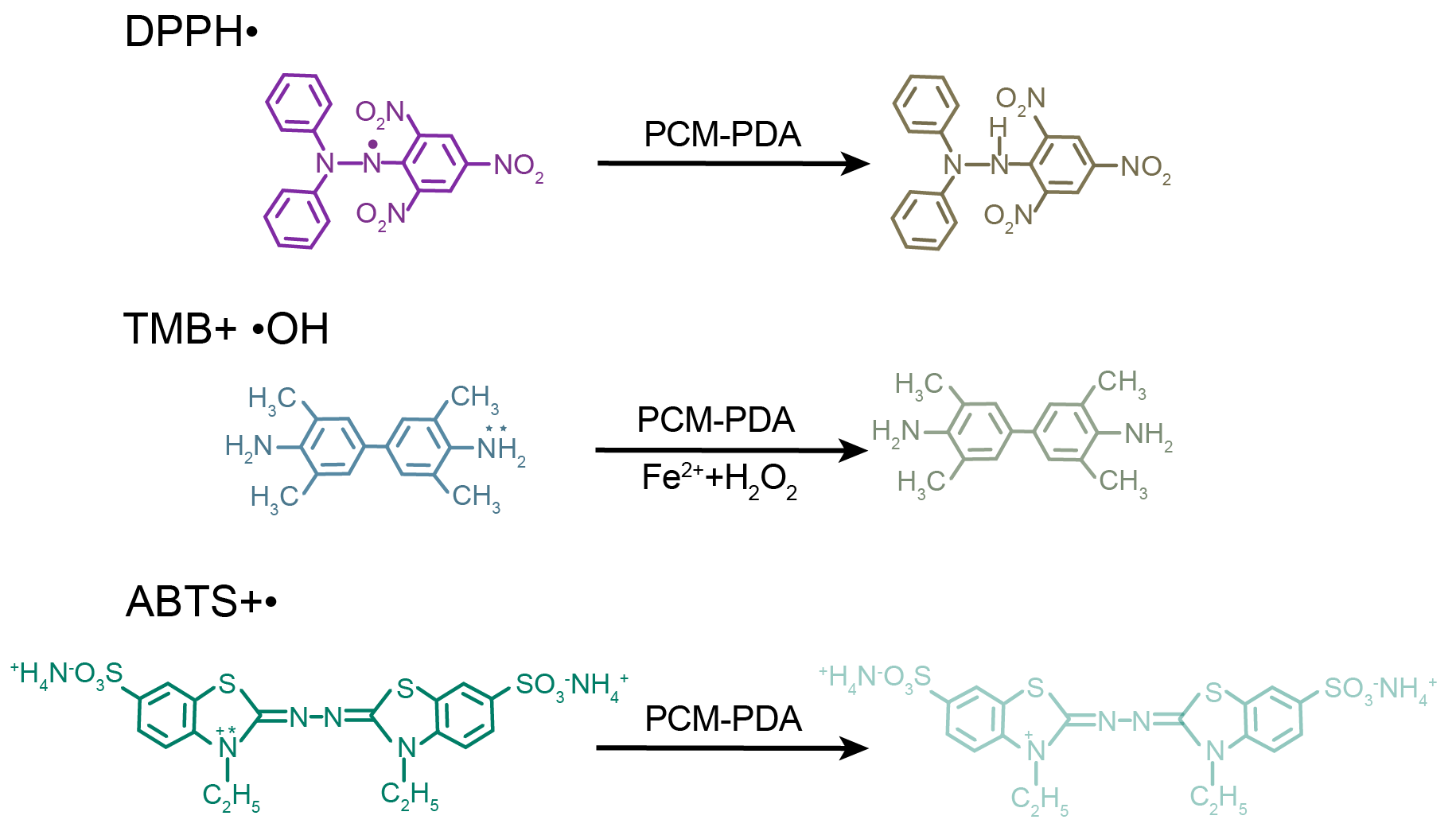


**Figure S2.** Schematic diagram showing how to measure the antioxidative activity of PDA nanocarriers.


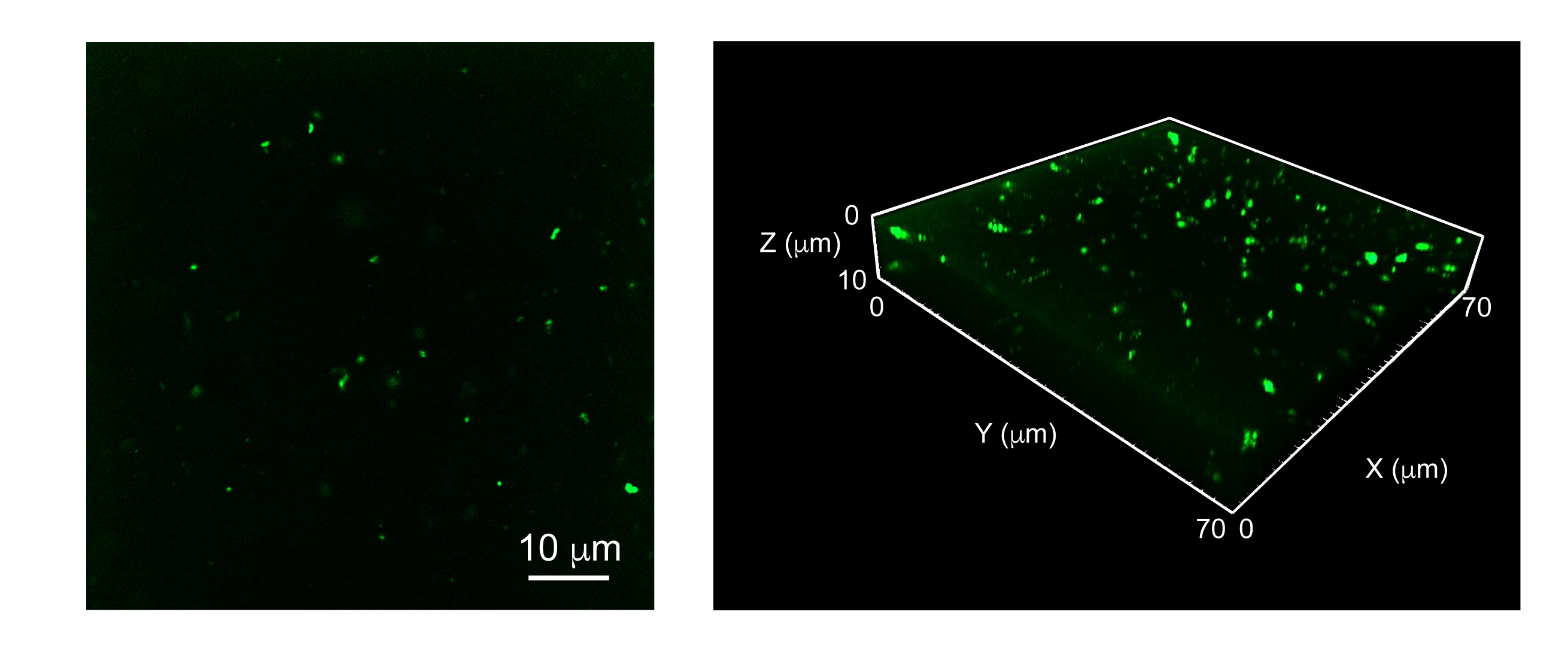


**Figure S3.** Subset (left) and Z-stack (right) confocal micrographs of PDA nanocarriers pre-loaded with FITC in the hydrogel.


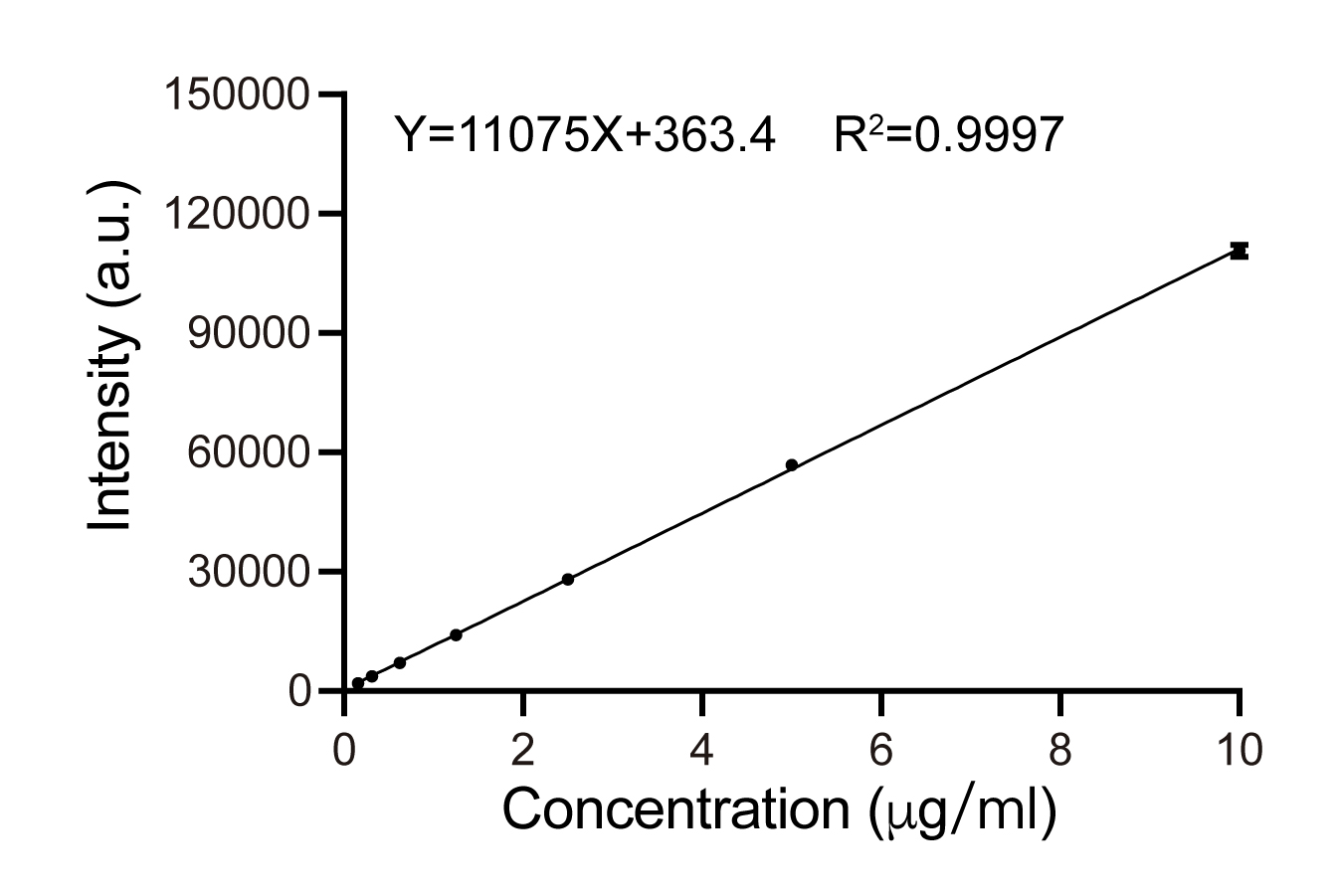


**Figure S4.** The fluorescence calibration curve of FITC. N=3


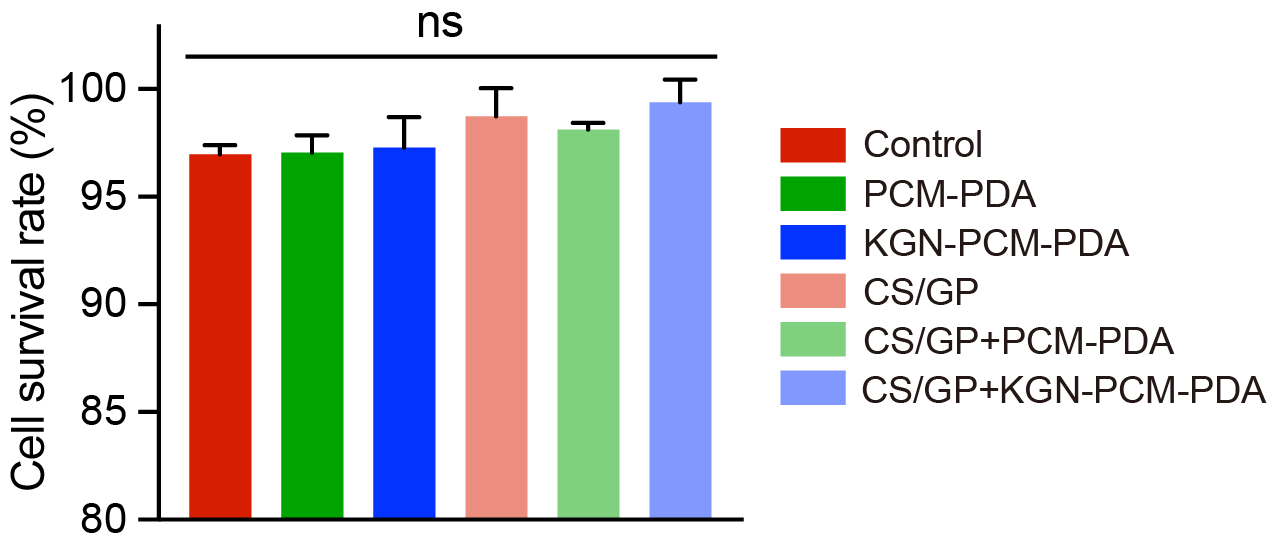


**Figure S5.** Statistics of cell survival rate based on live/dead staining. Data are presented as mean ± SD. At least 200 cells from three randomly selected images were counted to calculate the survival rate. One-way ANOVA with Tukey’s multiple comparisons test (ns, no significant).


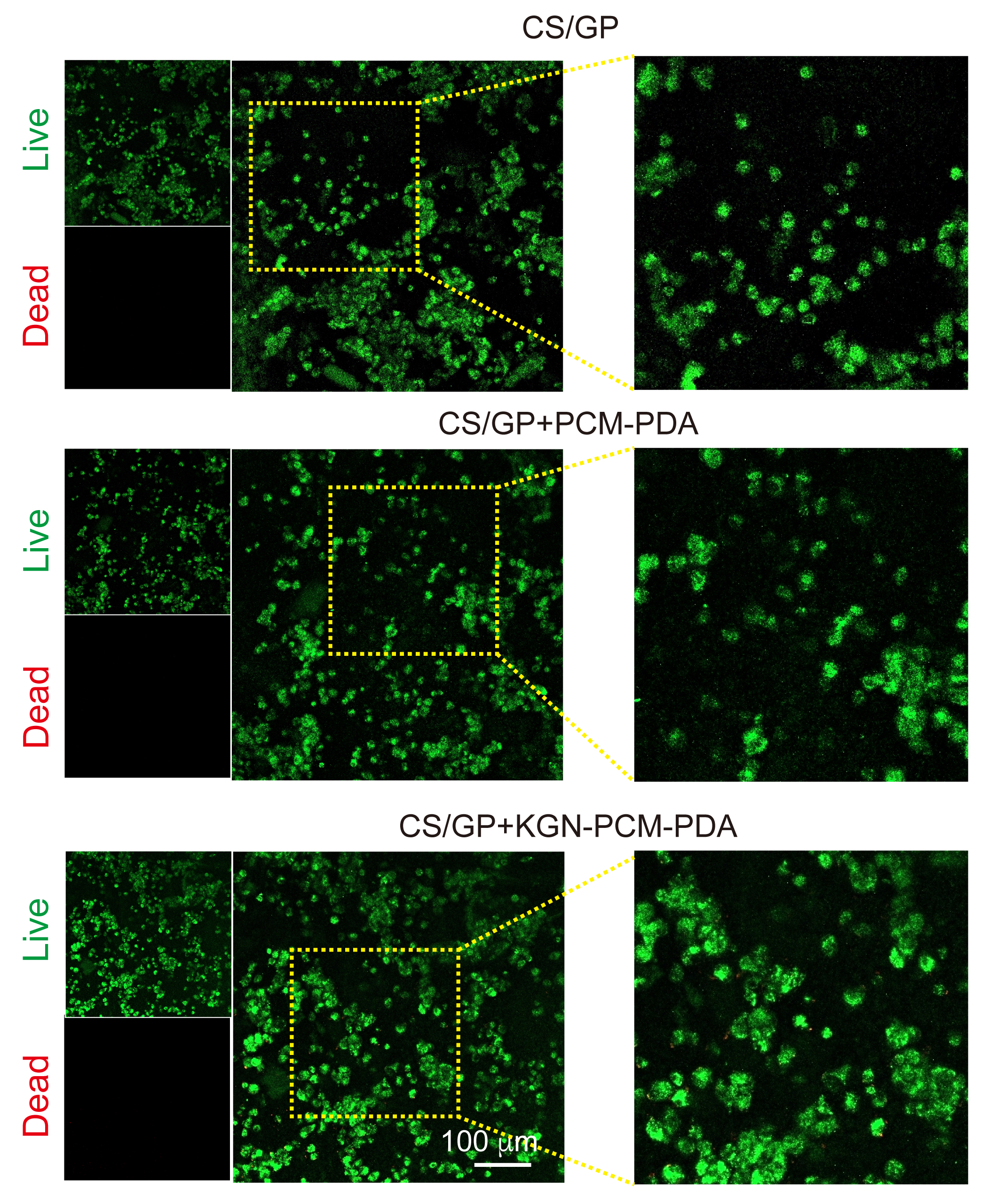


**Figure S6.** Z-stack superposed confocal micrographs after live/dead staining of the hMSCs cultured in the hydrogels for 48 h.


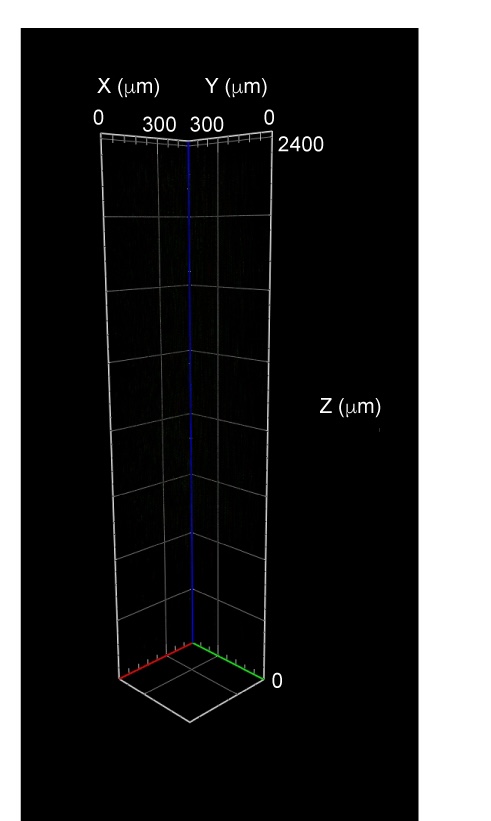


**Figure S7.** Z-stack confocal micrograph of the CS/GP hydrogel.


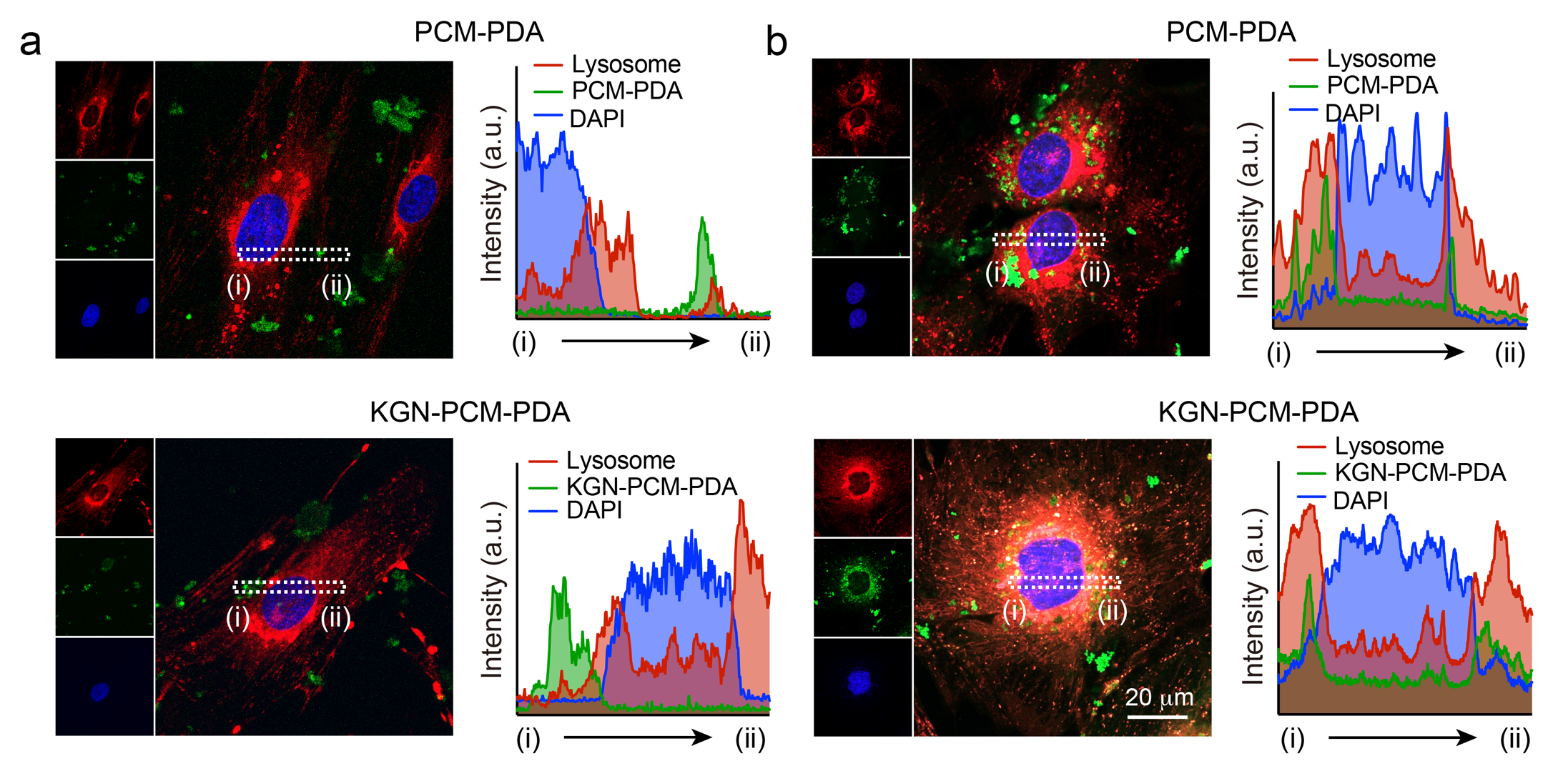


**Figure S8.** Confocal micrographs of the hMSCs after culture with PDA nanocarriers (green) for (a) 6 h and (b) 24 h, followed by lysosome (red) and DAPI (blue) staining and plots of the fluorescence intensities when moving from (i) to (ii).


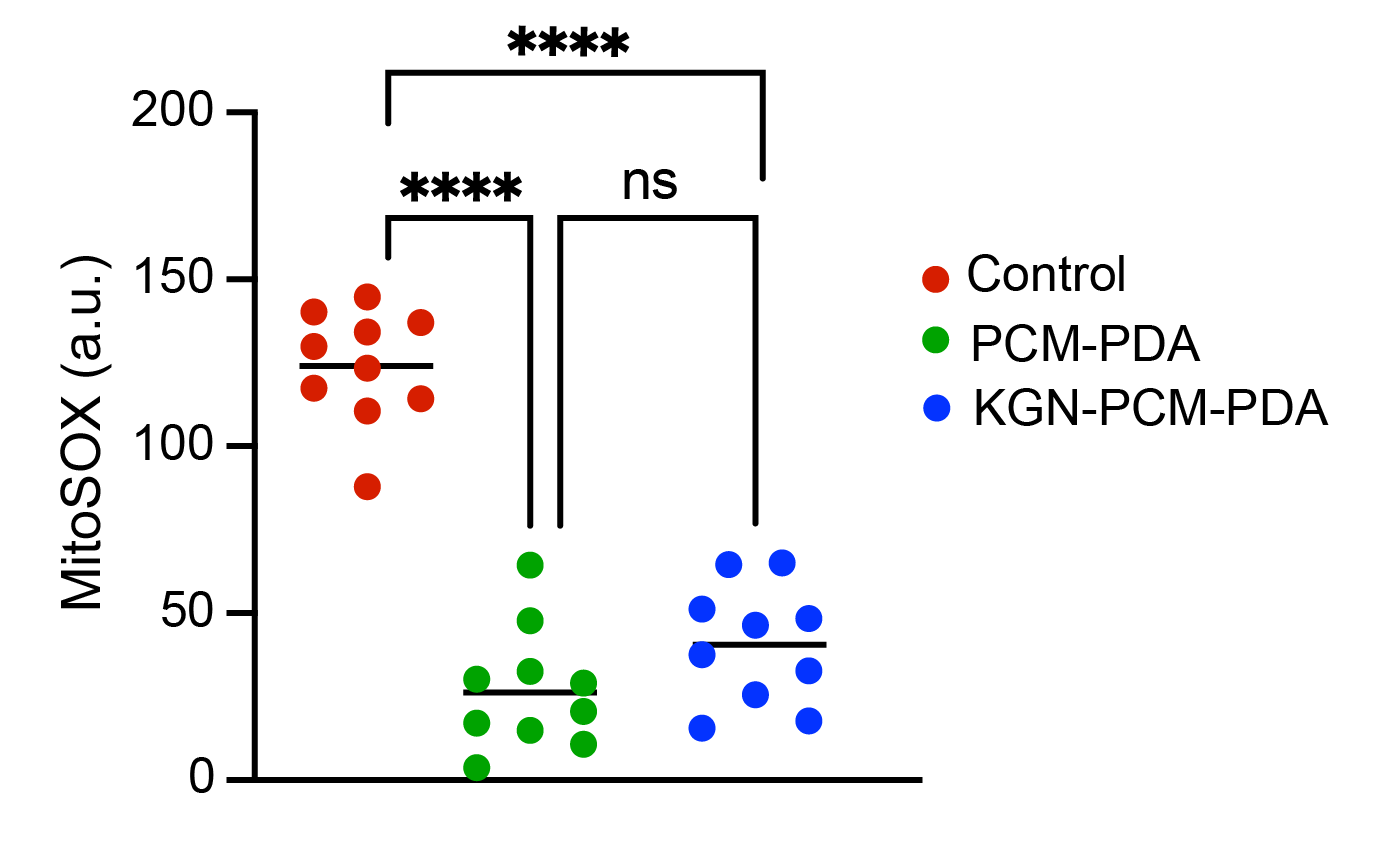


**Figure S****9.** Plots of the average fluorescence intensity of MitoSOX, with 10 random fields from three replications of each group being analyzed. One-way ANOVA with Tukey’s multiple comparisons test (^****^*p* < 0.0001).


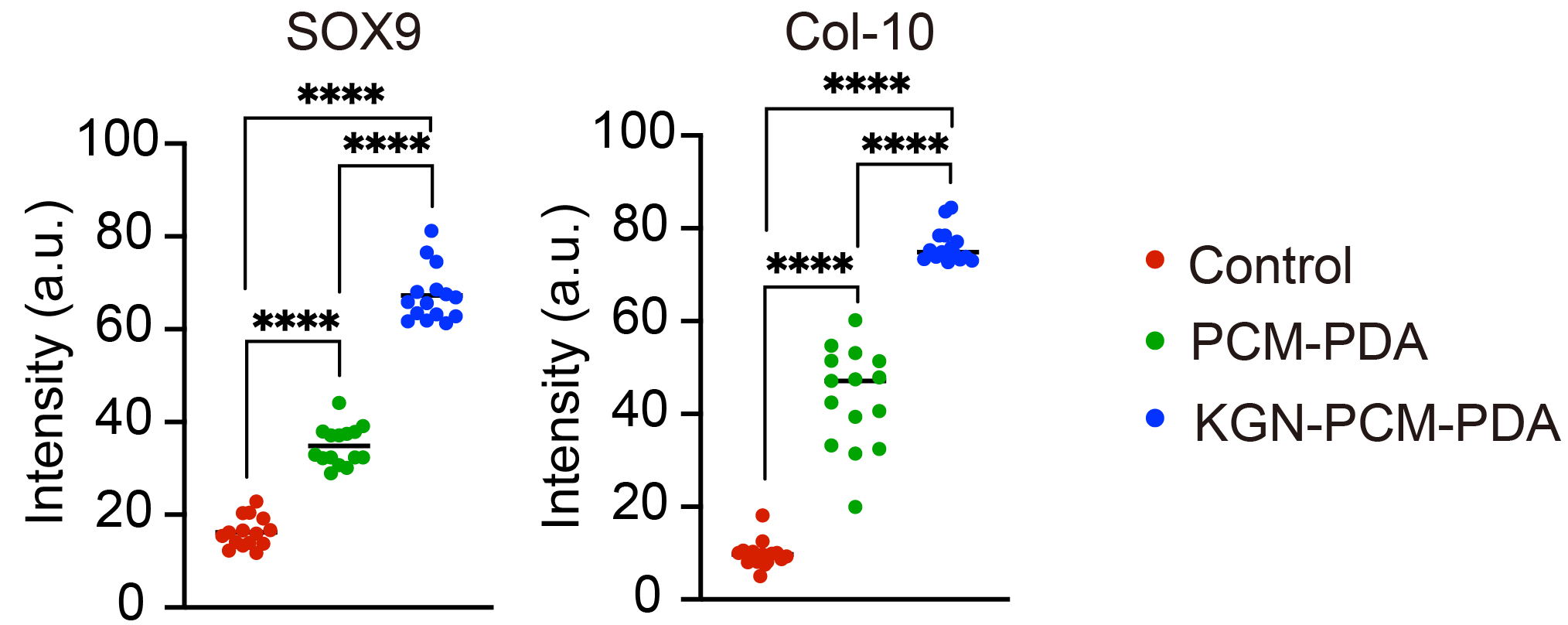


**Figure S10.** Plots of the average fluorescence intensity of SOX9 and Col-10 from the hMSCs after culture with two types of nanocarriers for 21 days, with 15 random fields from three replications of each group being analyzed. One-way ANOVA with Tukey’s multiple comparisons test (^****^*p* < 0.0001).


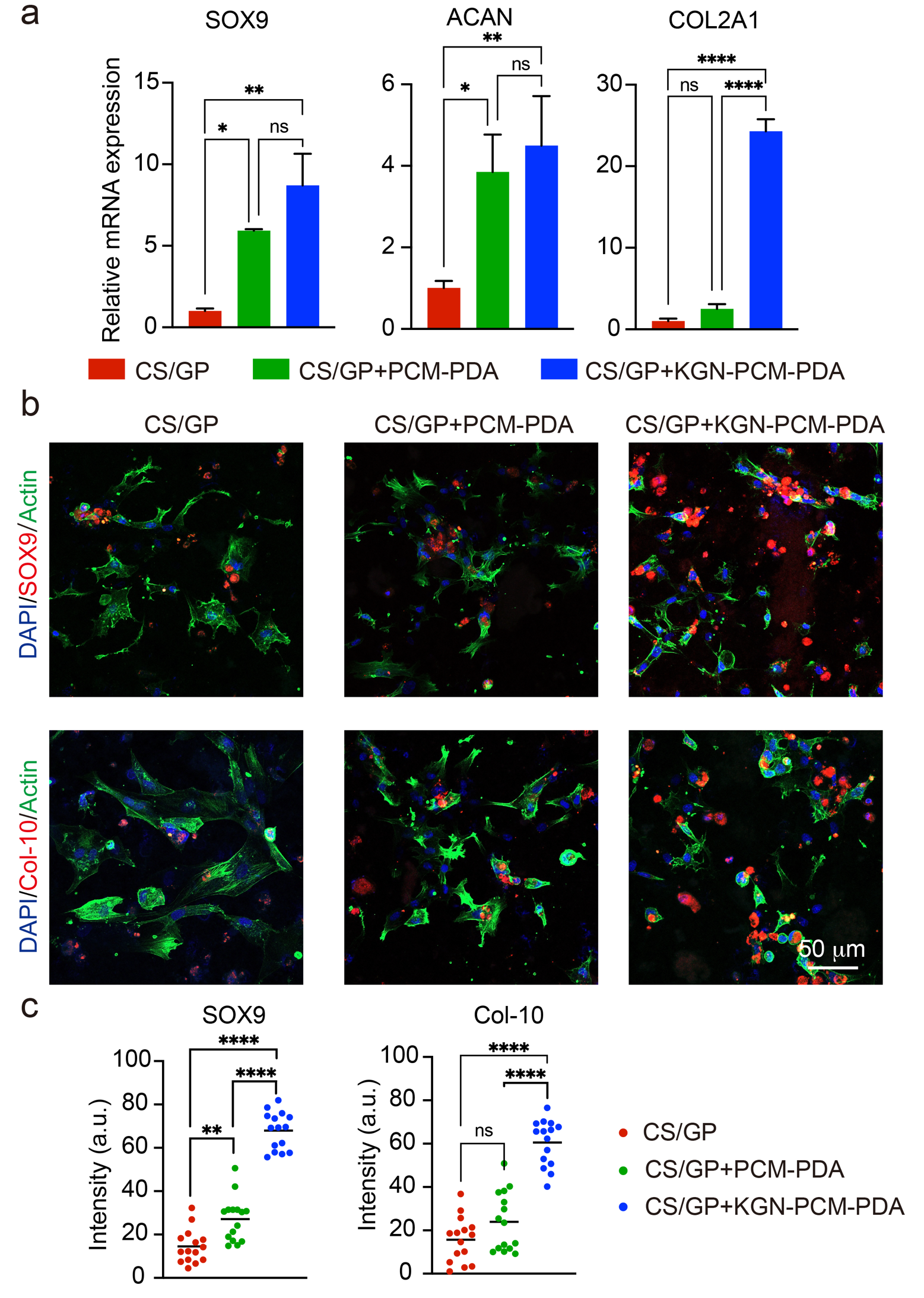


**Figure S11.** (a) RT-qPCR analysis of the hMSCs cultured in hydrogels for 14 days. N=3. One-way ANOVA with Tukey’s multiple comparisons test (^*^*p* < 0.05, ^**^*p* < 0.01, and ^****^*p* < 0.0001). (b) Confocal micrographs of the hMSCs culture in hydrogels for 21 days, followed by immunofluorescence staining. (c) Plots of the average fluorescence intensity of SOX9 and Col-10 based on (b). We analyzed 15 random fields from three replications of each group. One-way ANOVA with Tukey’s multiple comparisons test (^**^*p* < 0.01 and ^****^*p* < 0.0001).


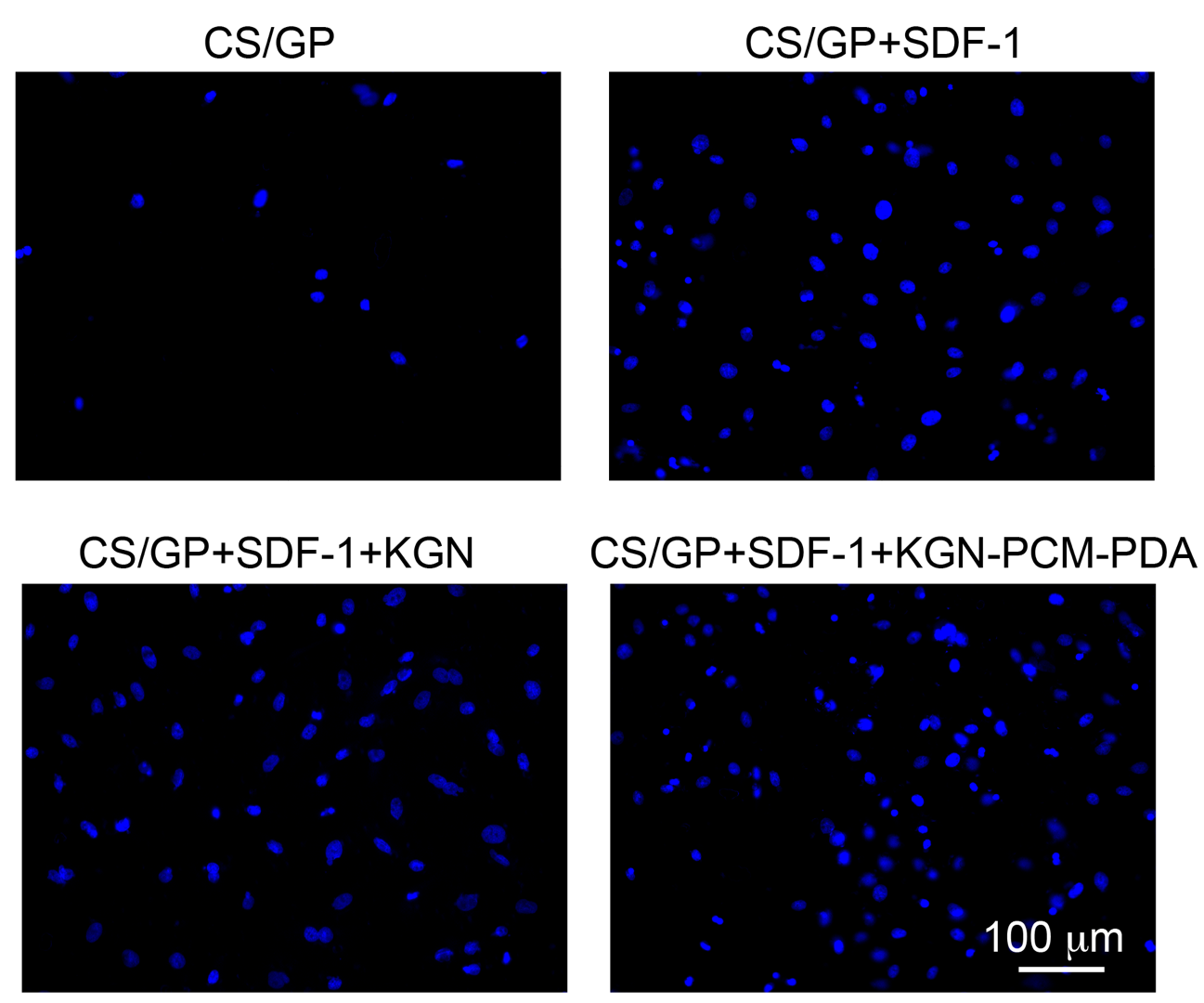


**Figure S12.** Fluorescence micrographs of the hMSCs residing on the basal side of the microporous membrane after culture for 12 h, followed by DAPI staining.


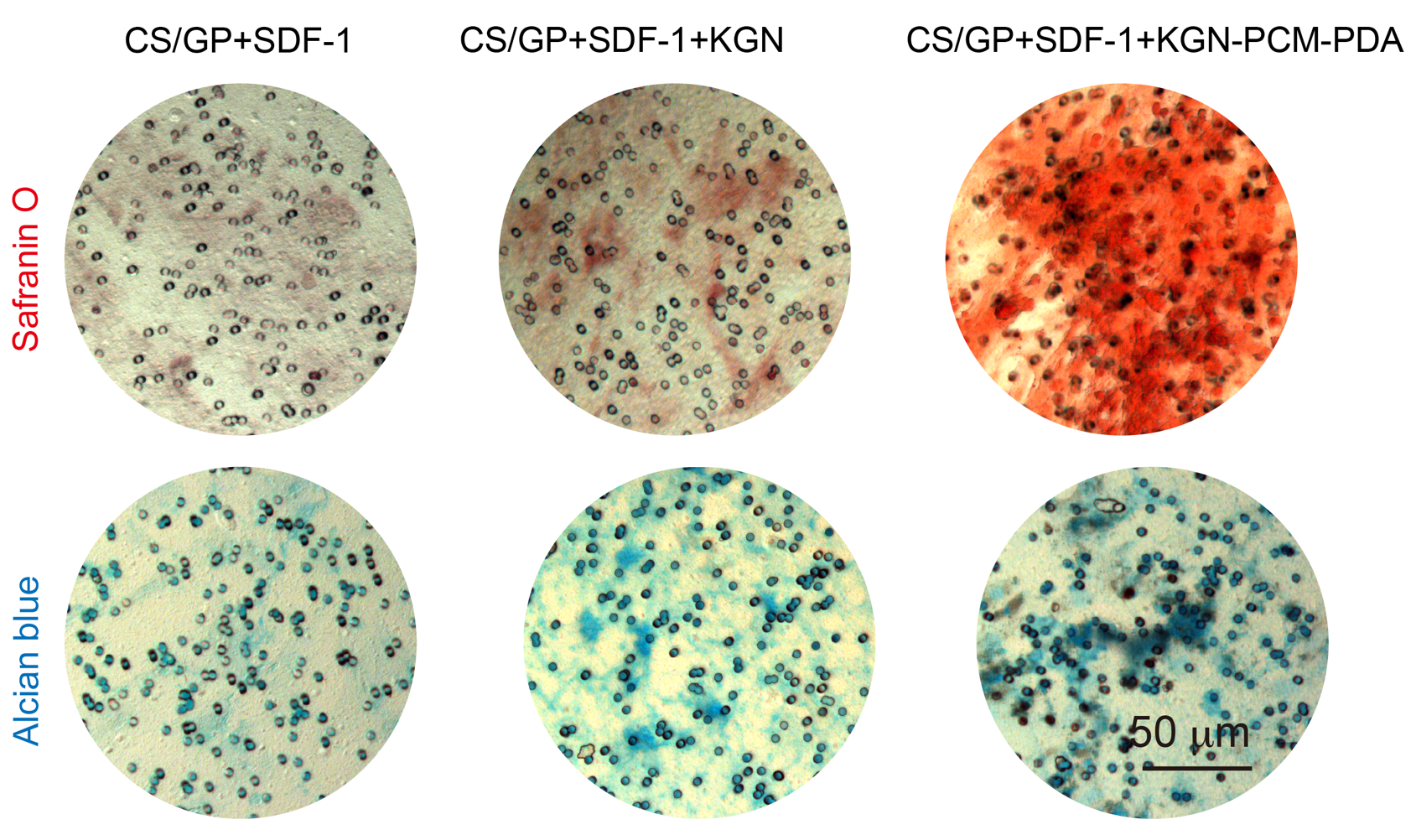


**Figure S13.** Optical micrographs of the hMSCs residing on the basal side of the microporous membrane after culture for 21 days, followed by safranin O and alcian-blue staining.

**References**

[1] J. Qiu, Y. Shi, Y. Xia, *Adv. Mater.* **2021**, *33*, 2104729.
